# Treatment of Steroid and Cyclosporine-Resistant Idiopathic Nephrotic Syndrome in Children

**DOI:** 10.4061/2011/930965

**Published:** 2011-09-22

**Authors:** A. A. Nikibakhsh, H. Mahmoodzadeh, M. Karamyyar, S. Hejazi, M. Noroozi, A. A. Macooie

**Affiliations:** ^1^Urology-Nephrology and Transplantation Research Centre, Department of Paediatric Nephrology, Urmia University School of Medicine, Iran; ^2^School of Medicine, Urmia University, Iran

## Abstract

Steroid-resistant nephrotic syndrome (SRNS) in children carries a significant risk of progression to end-stage renal failure (ESRF). We report a two-step protocol adapted in children with SRNS. Thirty-seven SRNS were treated with cyclosporine A (CyA) in association with prednisolone (alternate day) for 6 months (first-step treatment). Twelve patients (32.4%) went into complete remission, and 2 (5.4%) got partial remission. The other 23 cases who were steroid and CyA resistant entered a second-step treatment with withdrawing steroids, with CyA (5 mg/kg/day) in association with mycophenolate mofetil (MMF) 30 mg/kg/day for 6 months. Complete remission was observed in 11 cases (47.82%) and partial remission in 2 cases (8.7%). After two steps of treatment, 27/37 children went into total remission. In steroid and CyA-resistant INS, the association of MMF with CyA was able to induce remission in about half cases without relevant side effects.

## 1. Introduction

 Idiopathic nephrotic syndrome (INS) includes minimal change disease (MCD), focal segmental glomerulosclerosis (FSGS), and diffuse mesangial proliferation (DMP). Patients with MCD usually have excellent prognosis, but patients with FSGS and DMP often have hypertension, hematuria, and progress to renal failure [1–4]. Steroid dependence or resistance seems to have increased over the last decades, but the treatment of difficult cases of INS, mostly due to FSGS, is still a challenge [5–7]. A number of medications such as (MP) [7], cyclophosphamide [8], vincristine [9], MMF, and Cy A [10, 11] have been used with varying result. Recent reports have showed remission rates ranging from 20% to 70% using these drugs in SRINS [10, 11]. But serious side effects of these medications such as nephrotoxicity, hypertension, hirsutism, arrhythmias, psychosis, growth retardation, and severe infections remain a major problem [7–10]. Hence, the search for a rescue therapy in difficult cases of INS is needed in order to avoid unnecessary exposure to toxic drugs. An optimal combination of immunosuppresive agents to reverse resistance and to reduce the undesirable effects of high dose and prolonged administration of drugs is still a challenge (11, 4). Moreover, there are some reports supporting the idea that MCD, DMP, and FSGS are separate diseases because of different response rate to corticosteroid and the histologic findings offer data of prognostic significance [12]. Therefore, in many centers, renal biopsy is recommended according to the rationale that the intensity of immunosuppression may have to be adapted to the underlying findings in renal biopsy for the patients who do not respond to steroids [11–13]. 

In this study, patients with SRNS were treated in a first step protocol with a combination of Cy A (5 mg/kg/day) in association with prednisolone (1 mg/kg on alternate day) for 6 months. In resistant cases, prednisolone was withdrawn and Cy A (5 mg/kg/day) in association with MMF (30 mg/kg/day) was adapted as second step for 6 months. 

## 2. Methods and Materials

Patients admitted to our hospital between October 2002 and April 2008 fulfilling the entering criteria of INS (proteinuria >50 mg/kg/24 hr, serum albumin <3 g/dl, total cholesterol >250 g/dl, and edema) were included in this study. Children were treated with prednisolone 2 mg/kg/day for 4 weeks in case of remission, for 6 weeks in case of no remission.

The following criteria were adopted for assessing the patient's response to medication:

- complete remission: serum albumin ≥2.5 g% and absence of proteinuria or proteinuria <4 mg/kg/day,
- partial remission: serum albumin ≥2.5 g% and 50% reduction of initial proteinuria (<50 mg/kg/day),
- no response: persistence of hypoalbuminemia (<2.5 g%) and nephrotic proteinuria (≥50 mg/kg/day).

Patients with INS, older than 1 year and younger than 14 years old, without hypertension, macroscopic haematuria which went into remission with prednisolone (2 mg/kg/day) by 6 weeks, considered as SSNS and did not undergo kidney biopsy.

Patients who did not reach remission after 6 weeks of prednisolone (SSNS) underwent kidney biopsy which was examined by light microscope alone, since immunofluorescence and electron microscope were not available.

First-step protocol for SRNS was the following: Cy A (5 mg/kg/24 hr) and prednisolone (1 mg/kg/day) every other day for 6 months. If remission was obtained 3 month after complete remission, prednisolone was discontinued.

Second-step protocol for patients who did not go into remission on the first step: treatment Cy A 5 mg/kg/day and MMF 30 mg/kg/day were given for 6 months with withdrawing Prednisolone. If complete remission was obtained, 3 month later CyA was tapered. With any relapse during tapering phase, we go back again to previous dose of cyclosporine. 

Patients were monitored every 2 weeks for first 2 months, monthly for the next 6 months and every 2 months thereafter.

Data analyzed by chi square test with using two methods; Likelihood Ratio Test and Fisher's exact test. The *P* value was estimated Mont Carlo and exact distribution and value <0.05 was considered significant. In this study we used SPSS software version 16.

## 3. Results

One hundred patients with INS were investigated. A few cases were lost from followup (3 cases), 2 had congenital nephrotic syndrome, one had nephrotic syndrome associated with systemic lupus erythematosus, and 4 with membranoproliferative glomerulonephritis were not included in the study protocol. The remaining 90 patients considered suffer from INS entered the protocol.

Thirty-one children (34.4%) were female and 59 (65.6%) male (male to female ratio 1.9 : 1). 

In this study, 53 children (58.9%) responded well to steroid therapy, and thus did not undergo renal biopsy. Thirty-seven cases (41.1%) were resistant to steroid therapy and underwent renal biopsy and included DMP 11 cases (12.2%), FSGS 10 cases (11.1%), and MCD 16 cases (17.8%). In this group 15 (40.5%) children were <4 years old (y.o), 12 (32.5%) were between 4–10 (y.o), and 10 (27%) were >10 (y.o). The most common age group was <4 years, and the mean age of the patients was 7.2 ± 3.76 years (Table 1).

 Of 37 SRNS, 12 cases (32.4%) completely responded to first-step protocol and 2 cases (5.4%) partially responded to this regimen (Table 2). Steroid therapy was tapered in 12 children with complete remission and eventually discontinued in 8 children (7 MCD and 1 DMP) 3 months after complete remission.

Other 23 children remained steroid and CyA resistant and entered the second-step treatment. Eleven cases (47.82%) completely responded to CyA in association with MMF (second step), and 2 cases (8.7%) partially responded to this regimen (Table 2). We slowly tapered CyA (1 mg/kg/month) in 11 cases 3 months after the complete remission. All cases maintained remission with CyA (3 mg/kg) in association with MMF (30 mg/kg/day). When CyA was decreased to 2 mg/kg, 7/11 cases (6 cases with DMP and 1 cases with FSGS) completely and 2/11(MCD) partially relapsed.

The highest rate of response to steroid was in the age croup of 4–10 years (49%) and the highest rate of steroid resistance was in the smaller children <4 years old (41%).

Significant difference in complete response to combination therapy was found among the three groups (MCD, FSGS, and DMP) (Tables 3 and 4). The highest response rate to CyA and prednisolone was observed in patients with MCD (37.8%). The highest response rate to MMF and CyA was seen in DMP cases (56.5%). Ten cases were resistant to all drug categories.

Side effects of treatment were striae in 11, hypertrichosis in 9 and gingival hyperplasia in 5, and cataract in 3 patients in the end of first-step protocol. In the second-step protocol side effects of treatment were diarrhea in 3, abdominal pain in 4, and mild anemia in 3 patients.

## 4. Discussion

Children with SRNS have been treated with various medication, including CyA, MMF, cyclophosphamide, and azathioprine. In recent studies, the rate of complete remission after using different immunosuppressive agents varies from 30% to 84%, depending on the treatment protocols [11, 14, 15]. In a study by Jameela A. Kari, twenty of children with SRNS (65%) achieved partial (6) and complete (14) remission. In this report, 16 children was treated with cyclophosphamide either oral or intravenous, and only 4 of them (25%) achieved remission. Seven children received oral chlorambucil, and only 2 of them (28.5%) got remission. Fifteen children received CyA, and 8 of them (53%) achieved remission. MMF was used in 5 children, and 2 (40%) of them attained complete remission [15]. However because of unfavorable side effects, variable efficacy and high relapse rate in the literature, the alkylating agents are not recommended for primary therapy in SRNS [4, 6, 15]. In another report with using CyA in association with prednisolone in SRNS for time, 75% of cases got remission [11].

In our study the total remission was 73/1% (sum of remission obtained within first and the second protocol steps). Difference in complete response to combination therapy between three groups (MCD, FSGS, and DMP) was statically significant (Tables 3 and 4). The highest response rate to cyclosporine A + prednisolone was seen in patients with MCD (37.8%), but the highest response rate to MMF + cyclosporine A was detected in DMP cases (56.5%) (Tables 3 and 4). Seven cases which were resistant to all drugs had FSGS in biopsy and Only 3 cases (30%) with FSGS got remission (by first and second step) see Tables 3 and 4.

It is possible that the response rates in SRNS to immunosuppressants may vary according to the type of glomerular lesion and unknown multiple factors. Comparison between FSGS data and MCD data by Ehrich et al. has supported the concept that the type of underlying glomerular lesion in idiopathic SRNS may influence the response to treatment [11]. 

In the present study, the highest remission rate to steroid therapy was observed in the age of 4–10 years, and the highest resistance rate to steroid was seen in children <4 years old (Table 2). Other studies indicate increasing frequency in SRNS especially in young children [2, 11].

 Regarding irreversible side effects of long-term use of steroid especially in young children (growth retardation, cataract, and striae), it is suggested to use other medication such as MMF. The use of MMF in glomerulonephritis is based in its anti-inflammatory, antifibrotic, antiproteinuric, and inhibitory effects to mesangial cell proliferation [16]. Recently, Li et al. have reported that MMF is tolerated and significantly decrease urinary protein excretion in children less than 2 years old with SRNS [17]. In a randomized controlled multicenter study the efficacy of MMF versus CyA in children with relapsing MCD was investigated. In this study, MMF was given at maximum dose of 2 gr/day and CyA at dose of 4-5 mg/kg/day (for 12 months). Although the relapsed rate of nephrotic syndrome was higher in the MMF group, but the renal function was better preserved and the side effects were less observed with MMF [18]. In order to avoid high doses and long-term use of corticosteroid, some studies recommend the association of CyA or MMF with low dose corticostroide. MMF seems to be effective at least in some steroid and CyA resistant INS, but there are not reports about using of combination of MMF with CyA in steroid and CyA resistant INS.

Considering favorable experiences with MMF such as less adverse effects than other immunosuppressive drugs, benefit effects in the treatment of many immunologically mediated renal diseases, and the antifibrotic and antiproliferative effects of this medication [17, 18], it is suggested that MMF in association with low-dose CyA (after remission) can be a promising treatment of difficult cases of steroid and CyA resistant INS. 

In our series of cases, FSGS was the most common type of renal lesion in cases resistant to all therapy regimens. Only 3/10 cases (30%) with steroid resistant FSGS achieved remission the using first treatment (1 case) and the second step (2 cases). 

In some of these cases, we cannot exclude genetic mutations in podocyte-specific genes wich may influence the response to treatment [19–21]. Other important flaw of our study is loss of immunofluorescence and electron microscopic finding in kidney biopsy which was examined by light microscope alone, since immunofluorescence and electron microscope were not available.

Sensitivity rate of INS to steroid therapy was 58.9% in our study and 70%–80% in other reports [22–24]. It could be because of treating sensitive cases in provinces and not to referring them to our center.

Patients were monitored every 2 weeks for first 2 months, monthly for the next 4 months, and every 3 months thereafter. There was no significant side effect, and no change in dose of medication was made.

In conclusion, MMF in associated with CyA-induced remission in significant number of steroid and cyclosporine-resistant NS and kept them in remission with low-dose CyA.

## Figures and Tables

**Table 1:** Demography of 37 corticosteroid resistant nephritic syndrome.

Number	37
Sex_Girl (%)	15 (41%)
Age (y)	
<4	15 (40.5%)
4–10	12 (32.5%)
>10	10 (27%)

**Table 2:** Response of Nephrotic Syndrome to combination therapy.

Medication	Yes			No	Total number
	Complete	Partial	Total		
First step treatment Cyclosporine A + perdnisolone	12	2	14	23	37
Second step treatment Cyclosporine A + MMF	11	2	13	10	23

**Table:** (a)

Histology	Response		Total
	Nonresponse	Response
MCD	6 (37.5%)	10 (62.5%)	16 (100%)
DMP	8 (72.4%)	3 (27.3%)	11 (100%)
FSGS	9 (90%)	1 (10%)	10 (100%)

**Table:** (b)

Statistical test	Monte Carlo*	Exact sig
Likelihood ratio = 8.519 d.f = 2	Sig (2-sided) = 0.021	Sig (2-sided) = 0.021
Fishers Exact test	Sig = 0.023*	*P* = 0.023

*Based on 1000 sampled tables.

**Table:** (a)

Histology	Response		Table
	Nonresponse	Response	
MCD	2 (33.3%)	4 (66.7%)	6 (100%)
DMP	1 (12.5%)	7 (87.5%)	8 (100%)
FSGS	7 (77.8%)	2 (22.2%)	9 (100%)

**Table:** (b)

Statistical test	Mont Carlo	Exact sig
Likelihood ratio = 8.291 df = 2	Sig (2-sided) =0.03*	sig (2-sided) = 0.021
Fishers exact test	Sig = 0.021*	*P* = 0.021

*Based on 1000 sampled tables.

## References

[1] Filler G, Young E, Geier P, Carpenter B, Drukker A, Feber J (2003). Is there really an increase in non-minimal change nephrotic syndrome in children?. *American Journal of Kidney Diseases*.

[2] Wang JG, Zhou ZL, Pan T, Li CC, Yang Q (2002). Integrated traditional Chinese and Western medicine for infantile nephrotic syndrome-150 cases report. *Shanghai Journal of Traditional Chinese Medicine *.

[3] McBryde KD, Kershaw DB, Smoyer WE (2001). Pediatric steroid-resistant nephrotic syndrome. *Current Problems in Pediatric and Adolescent Health Care*.

[4] Coppo R (2008). Non-steroidal and non-cytotoxic therapies for nephrotic syndrome. *Nephrology Dialysis Transplantation*.

[5] Yu L, Wen ZY, Hua SD (2002). Analysis of clinical characteristics in infantile nephrotic syndrome. *Chinese Journal of Practical Pediatrics*.

[6] Hodson EM, Willis NS, Craig JC (2008). Non-corticosteroid treatment for nephrotic syndrome in children. *Cochrane Database of Systematic Reviews*.

[7] Yorgin PD, Krasher J, Al-Uzri AY (2001). Pulse methylprednisolone treatment of idiopathic steroid-resistant nephrotic syndrome. *Pediatric Nephrology*.

[8] Tune BM, Kirpekar R, Sibley RK, Reznik VM, Griswold WR, Mendoza SA (1995). Intravenous methylprednisolone and oral alkylating agent therapy of prednisone-resistant pediatric focal segmental glomerulosclerosis: a long-term follow-up. *Clinical Nephrology*.

[9] Goonasekera CDA, Koziell AB, Hulton SA, Dillon MJ (1998). Vincristine and focal segmental sclerosis: do we need a multicentre trial?. *Pediatric Nephrology*.

[10] Mendizábal S, Zamora I, Berbel O, Sanahuja MJ, Fuentes J, Simon J (2005). Mycophenolate mofetil in steroid/cyclosporine-dependent/resistant nephrotic syndrome. *Pediatric Nephrology*.

[11] Ehrich JHH, Geerlings C, Zivicnjak M, Franke D, Geerlings H, Gellermann J (2007). Steroid-resistant idiopathic childhood nephrosis: overdiagnosed and undertreated. *Nephrology Dialysis Transplantation*.

[12] Segarra A, Vila J, Pou L (2002). Combined therapy of tacrolimus and corticosteroids in cyclosporin-resistant or -dependent idiopathic focal glomerulosclerosis: a preliminary uncontrolled study with prospective follow-up. *Nephrology Dialysis Transplantation*.

[13] Ejaz I, Khan HI, Javaid BK, Rasool G, Bhatti MT (2004). Histopathological diagnosis and outcome of paediatric nephrotic syndrome. *Journal of the College of Physicians and Surgeons Pakistan*.

[14] Del Rio M, Kaskel F (2008). Evaluation and management of steroid-unresponsive nephrotic syndrome. *Current Opinion in Pediatrics*.

[15] Kari JA, Halawani M (2010). Treatment of steroid resistant nephrotic syndrome in children. *Saudi journal of kidney Diseases and Transplantation*.

[16] Dubus I, Vendrely B, Christophe I (2002). Mycophenolic acid antagonizes the activation of cultured human mesangial cells. *Kidney International*.

[17] Li Z, Duan C, He J (2099). Mycophenolate mofetil therapy for children with steroid-resistant nephrotic syndrome. *Pediatric Nephrology*.

[18] Dorresteijn EM, Kist-van Holthe JE, Levtchenko EN, Nauta J, Hop WCJ, van der Heijden AJ (2008). Mycophenolate mofetil versus cyclosporine for remission maintenance in nephrotic syndrome. *Pediatric Nephrology*.

[19] Habashy D, Hodson EM, Craig JC (2003). Interventions for steroid-resistant nephrotic syndrome: a systematic review. *Pediatric Nephrology*.

[20] Ruf RG, Lichtenberger A, Karle SM (2004). Patients with mutations in NPHS2 (Podocin) do not respond to standard steroid treatment of nephrotic syndrome. *Journal of the American Society of Nephrology*.

[21] Gipson DS, Gibson K, Gipson PE, Watkins S, Moxey-Mims M (2007). Therapeutic approach to FSGS in children. *Pediatric Nephrology*.

[22] McBryde KD, Kershaw DB, Smoyer WE (2001). Pediatric steroid-resistant nephrotic syndrome. *Current Problems in Pediatric*.

[23] Bonilla-Felix M, Parra C, Dajani T (1999). Changing patterns in the histopathology of idiopathic nephrotic syndrome in children. *Kidney International*.

[24] Barnett HL, Edelmann CM, Greifer I (1981). The primary nephrotic syndrome in children. Identification of patients with minimal change nephrotic syndrome from initial response to prednisone. *Journal of Pediatrics*.

